# Cost Awareness of Anaesthetic Consumable Items Among the National Health Service (NHS) Staff and the Financial Impact on the NHS

**DOI:** 10.7759/cureus.63906

**Published:** 2024-07-05

**Authors:** Omkaar Divekar, Abhinav Kumar, Nandita Divekar, Rahul Kanegaonkar

**Affiliations:** 1 Trauma and Orthopaedics, St. George’s University, London, GBR; 2 Respiratory Medicine, Wrightington, Wigan and Leigh National Health Service (NHS) Foundation Trust, Manchester, GBR; 3 Anaesthesiology, Medway Maritime Hospital, Medway, GBR; 4 Ear, Nose & Throat, Canterbury Christ Church University, Canterbury, GBR

**Keywords:** medical education, anaesthetics, cost analyses, sustainable healthcare, health economics

## Abstract

Background

The financial burden of running the National Health Service (NHS) is high. Staff members should be aware of the cost of the equipment they use to enable efficient use of resources, reduce waste, and control spending. However, limited financial education at undergraduate and junior stages has contributed to relatively poor knowledge among healthcare workers at all levels. Anaesthetics is a speciality which uses a large amount of equipment; therefore, we aim to assess the cost awareness among staff for commonly used consumables. Furthermore, we aim to assess staff members’ attitudes towards the financial and environmental impact of the equipment they use and whether this would change their practice.

Methodology

An electronic survey was sent to staff members from the anaesthetic department of the Medway NHS Foundation Trust during a one-month period. Respondents were asked to estimate the cost of 19 commonly used anaesthetic consumables, with an estimate categorised as correct if it was within 20% of the actual cost. At the end of the survey, there were five questions for respondents to answer regarding the financial and environmental impact of their current healthcare practice and possible alternatives.

Results

There were 69 respondents within the anaesthetic department from a variety of roles. Overall, only 9.37% of items were estimated correctly, with cheaper items commonly being overestimated and more expensive items being underestimated. Overall, 60% of respondents said the cost of an item would influence their use. The overwhelming majority claimed that the environmental impact was a concern, and most would favour recyclable/reusable alternatives.

Conclusions

Cost awareness among anaesthetic staff for commonly used equipment is poor. More education and training are necessary in this area as limited knowledge of service costs restricts the ability to make cost-efficient choices which are needed in the current NHS.

## Introduction

The financial burden of running the National Health Service (NHS) is high, with expenditure in the NHS rising from £58.9 billion in 2008/2009 to £181.7 billion in 2022/2023 [1]. NHS spending per capita and as a percentage of GDP has also been rising over time [2]. Hospital-based care accounts for the largest proportion of NHS expenditure, amounting to over 50% of total NHS spending and has grown by 54.1% from 2008/2009 to 2016/2017 [1]. Increased life expectancy and access to healthcare mean this trend in increased health spending can also be seen globally [3,4]. With this high expenditure, there is a greater need to understand hospital expenses to run the service and control spending. NHS staff members frequently experience the effects of tightening budgets within the NHS but may not be aware of the costs of the care they provide. Therefore, it is important that staff should be informed and aware of the cost of equipment they use to enable efficient and effective use of resources, while not compromising the standard of care they provide to patients.

Previous studies have assessed staff members’ awareness of the cost of equipment they use. When a survey was distributed to staff in an otolaryngology department at McGill University and at Western University asking respondents to estimate the cost of commonly used products, they concluded that staff members had poor knowledge of the cost of items, with fewer than 10% of respondents being able to accurately estimate the costs (an estimate was considered accurate if it was within 50% of the true cost) of at least half the products [5]. A similar study was performed in an orthopaedic department at a UK district general hospital in which a survey was distributed to staff and patients in orthopaedic wards and clinics. This study concluded that both staff and patients had a generally poor understanding of the cost of items and services within the NHS [6]. Furthermore, both the otolaryngology and orthopaedic department studies concluded from their respondents that a greater cost awareness of items would influence practitioners’ approach to their clinical behaviour and practice. They hypothesise that greater cost awareness can lead to cost savings through reduced waste.

It would be reasonable to expect that greater cost awareness within the NHS may be correlated with seniority, as job experience means individuals would have a better understanding of the health service and be more likely to make decisions regarding equipment choice/procurement. However, a study of UK surgical junior doctors and consultants in a variety of specialities found that among all grades and specialities, the knowledge of device costs was poor, with no statistically significant difference between training levels [7]. This highlights a lack of education in health economics for trainees and a subsequent lack of knowledge among senior doctors. A study assessing the cost awareness of surgical equipment among 326 surgeons in Ireland found similar results, with no significant difference in awareness between junior and senior surgeons [8]. When these surgeons were questioned further regarding health economics education, only 5.7% reported undergraduate teaching on the topic, and only 7.8% felt they had sufficient training in health economics.

While the choice of equipment used or discarded is dictated by treatment and procedure protocols, operator choices cannot be overlooked, and staff members can modify their behaviour to reduce equipment wastage. A study of 58 procedures at a neurosurgical department at the University of California found that there was on average $653 worth of preventable operating room waste (unused items) per case, with 85% of operating room waste coming from disposable items [9]. These results are related to a specific neurosurgical department, therefore, it may not be appropriate to extrapolate this to every operating department or speciality. Interestingly, this study also concluded that there was no significant difference in waste when considering the operators’ years of experience, further strengthening the idea that financial education has been lacking at all levels and greater cost awareness is not guaranteed with more healthcare experience.

The anaesthetic speciality is highly technical and involves using large amounts of consumable equipment [10]. Therefore, our study aims to assess current cost awareness within the anaesthetic speciality and if there is any improvement compared to previous studies. Furthermore, while it is desirable to cut costs and reduce waste, it is important to assess the environmental impact of healthcare decisions. Using recyclable/reusable equipment can offer a solution to equipment wastage in anaesthetics and can be more environmentally friendly than using single-use items. To understand the attitudes towards the cost of equipment and the environmental impact of consumable use, this study aims to survey staff members’ opinions on these issues and if they would be amenable to changing their practices.

This article was previously presented in oral and poster format at the British Association for Physicians of Indian Origin (BAPIO) national conference held in Manchester in 2023. The abstract was published in BAPIO’s journal, The Physician, on December 26th, 2023.

## Materials and methods

This study was approved by the Research and Innovation Department of East Kent Hospitals, with this survey forming part of a regional study of NHS staff members from a variety of specialties. To gather responses from staff, an electronic survey was generated using Microsoft Forms and sent to staff members from the anaesthetic department of the Medway NHS Foundation Trust. At the beginning of the online survey, a statement outlined that participants would need to provide demographic data, to estimate the cost of commonly used medical items, and, lastly, to explore their views surrounding the environmental impact of medical equipment. Participants were informed that all data provided would be anonymised and that it would be used in our study to understand how medical students perceive the cost of the medical equipment they use. In this way, consent was formally obtained from each survey respondent.

Responses were only recorded if the survey was completed during a one-month period from October 27th to November 28th, 2022, and if the staff member was working (in any medical role) within the anaesthetic department. Using these criteria, 68 responses were included in our study for data analysis. We also ensured there were no duplicate responses from the collected surveys. To ensure anonymity no identifiable data were stored and responses were collated on a secure spreadsheet. The methodology of this survey was based on previous studies that assessed medical personnel’s awareness of the cost of items that they use [5].

The survey first recorded basic demographic data such as sex, age and job role. Respondents were then asked to estimate the cost in pounds and pence of 10 commonly used disposable surgical items, followed by nine commonly used disposable anaesthetic items (see Appendix), with illustrations of the items also shown. Items 1-10 represent commonly used surgical items, and items 11-19 represent commonly used anaesthetic items. The actual cost of the items was taken from the East Kent Hospital procurement department using a standard procurement site (supplychain.nhs.uk). An estimate was categorised as correct if it was within 20% of the actual cost, which is in keeping with the methodology of previous studies in this area [7].

To further delineate the attitudes of staff regarding the cost of consumable items and their impact on the environment, at the end of the survey there were five yes/no questions for respondents to answer: (1) Should the cost of an item influence its use? (2) Is the environmental impact of healthcare of concern to you? (3) Should innovators consider the environmental impact of products during the development of devices? (4) Should the environmental impact of a product/device influence the clinical use of that product/device? (5) If two devices have similar benefits, but one is recyclable/reusable, would this influence your practice?

## Results

**Figure 1:**
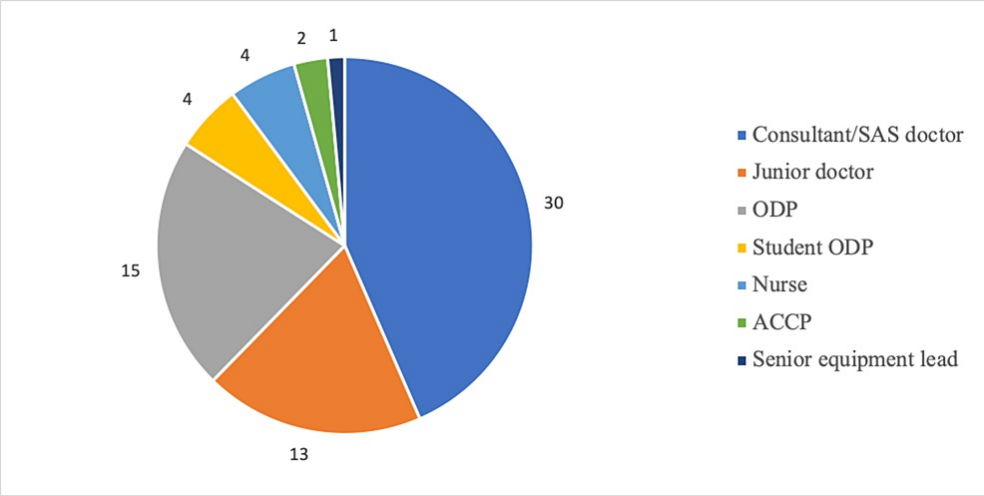
Survey respondents categorised by job title. SAS = speciality and associate specialist; ODP = operating department practitioner; ACCP = advanced critical care practitioner

There were 69 respondents to the survey from a variety of roles within the anaesthetic department (Figure 1). A link to complete the survey was sent to the entire anaesthetic department via email; however, unfortunately, as the exact number of people it was sent to is unknown, the overall survey response rate was not calculable. Most responses were from doctors of all levels, which included those who worked in anaesthetics or surgery. The other respondents included operating department practitioners (ODPs), student ODPs, nurses, advanced critical care practitioners (ACCPs), and a senior equipment lead.

Table 1 shows the list of items with the median estimate of the responses for each item; the median estimate divided by their actual cost to determine the degree of under/overestimation of the estimates; and the percentage of correct responses (estimates within 20% of actual cost). Across all the items, 9.37% of the estimates were correct. The item’s price that was most correctly estimated was the ‘10 mL syringe’ by 15 (21.7%) respondents, followed by the ‘spinal needle’ by 14 (20.3%) respondents. The least correctly estimated items were the ‘package of 2 4 × 4 sterile sponges’, the ‘1 L bag of intravenous normal saline’, the ‘disposable sterile surgical gown’, and the ‘size 8 cuffed oral endotracheal tube’, with only two (2.9%) respondents estimating correctly.

**Table 1:** List of items with their actual cost and the median estimate of the survey responses recorded.

Item	Actual cost (GBP)	Median estimate (GBP)	Median estimate/Actual cost	% of respondents within 20% of the actual cost
One (1) 15-blade scalpel	0.25	2.0	8.00	5.8
One (1) 3-0 Vicryl (non-rapide) suture	1.97	4.5	2.28	15.9
One (1)4-0 Prolene suture	2.98	4.0	1.34	8.7
One (1) 10 mL syringe	0.88	0.5	0.57	21.7
One (1) package of 2 4 × 4 sterile sponges	0.31	1.5	4.84	2.9
One (1) 1 L bag of intravenous normal saline	0.70	3.0	4.29	2.9
One (1) vial of 1.2 g intravenous co-amoxiclav	1.06	4.0	3.77	5.8
One (1) pair of standard sterile surgical gloves	1.03	2.1	1.99	17.4
One (1) box of fifty (50) surgical masks	2.58	5.3	2.05	11.6
One (1) disposable sterile surgical gown	1.36	6.7	4.93	2.9
One (1) size 4 transparent anaesthetic mask	0.72	3.0	4.17	4.3
One (1) size 8 cuffed oral endotracheal tube	0.61	5.0	8.20	2.9
One (1) size 4 disposable laryngoscope blade	2.76	5.0	1.81	10.1
One (1) disposable laryngoscope blade and handle	38.40	10.0	0.26	4.3
One (1) size 4 Guedel™ airway	0.22	1.9	8.41	7.2
One (1) disposable non-invasive blood pressure (NIBP) cuff	3.30	5.0	1.52	17.4
One (1) 50 mL plastic syringe	0.27	1.0	3.70	7.2
One (1) heat and moisture exchanger (HME) filter	0.79	2.0	2.53	8.7
One (1) spinal needle	6.24	4.0	0.64	20.3

**Figure 2:**
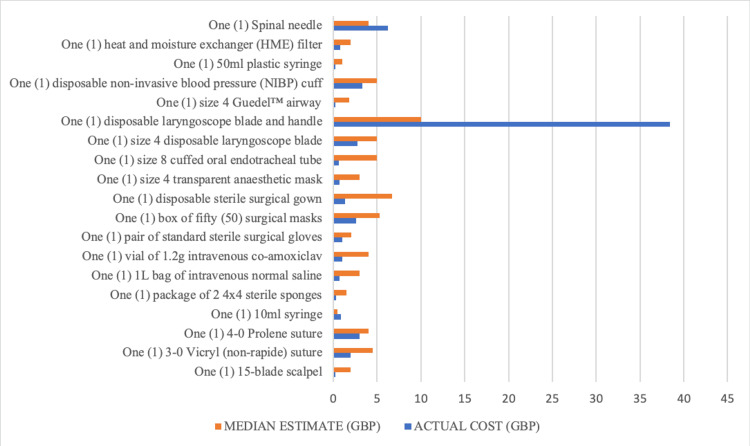
Graph comparing the actual cost and median estimated cost of the consumable items.

As demonstrated in Figure 2, for most items, the median estimate was higher than the actual cost. The only exceptions where the median estimate underestimated the actual cost were the ‘10 mL syringe’, the ‘disposable laryngoscope blade and handle’, and the ‘spinal needle’. The items with the most accurate median estimate were the ‘4-0 Prolene suture’ (median estimate/actual cost of 1.34), and the ‘spinal needle’ (median estimate/actual cost of 0.64). The item with the greatest overestimation of the cost was the ‘size 4 Guedel airway’ (median estimate/actual cost of 8.41), and the item with the greatest underestimation was the ‘disposable laryngoscope blade and handle’ (median estimate/actual cost of 0.26).

Table 2 shows the responses to the five yes/no questions at the end of the survey. For question 1, the responses were mixed; however, 40 (58%) respondents believed the cost of an item should influence its use compared to 29 (42%) who did not. For questions 2-5, most respondents answered yes, indicating that the environmental impact of the items and whether they are reusable/recyclable are of more importance to staff members than the cost of the items themselves.

**Table 2:** Responses to the questions regarding the use of surgical and anaesthetic items.

	Yes	No
Should the cost of an item influence its use?	40	29
Is the environmental impact of healthcare of concern to you?	65	4
Should innovators consider the environmental impact of products during the development of devices?	68	1
Should the environmental impact of a product/device influence the clinical use of that product/device?	54	15
If two devices have similar benefits, but one is recyclable/reusable, would this influence your practice?	65	4

## Discussion

These results indicate that practitioners in anaesthesia have a poor knowledge of equipment costs. This is in keeping with previous studies that surveyed cost awareness in anaesthetic departments [11-13]. Overall, 9.37% of estimates were correct, which is similar to previous studies in which less than 10% of estimates were correct and demonstrates a repeated pattern of financial illiteracy [5,7]. Furthermore, cheaper items were commonly overestimated and more expensive items were underestimated, which is again seen in previous studies assessing equipment cost awareness [8,14]. The results ultimately show there has been no significant improvement over the years in cost awareness among healthcare and anaesthetic staff.

It is important to stress that individual choices will have spending implications, and the economic use of goods and services contributes to NHS sustainability and ultimately reduce the financial burden. In this study, 58% of respondents stated that the cost of an item should influence its use. This question may be interpreted by respondents as choosing lower quality items to cut costs; however, the purpose was to elicit practitioners’ willingness to change their practice given more financial information. One study that assessed surgical practitioners’ cost awareness found that 69% believed the cost of the items would influence their practice, and another study found that 82% of respondents felt that greater information would change their use of consumables [5,8]. Alongside our results, this highlights that there is a general willingness to understand health economics better among the medical community.

Limited knowledge of equipment and service costs restricts a practitioner’s ability to make cost-efficient choices and more education in this is justified given the current financial climate in the NHS. Current medical education is deficient in health economics teaching, with variable amounts of teaching across UK universities [15]. Healthcare workers feel undertrained in health economics, and as we have seen in our study, their practice might change if they were more cost-aware. Targeted training to different specialities (not just anaesthesia) on the costs of their commonly used equipment can be used to generate a fully integrated health system focussed on waste reduction and cost containment. For instance, in surgical specialities, when surgeons were informed of the costs of the equipment used in their operating theatres during cases, there was a significant reduction in their personal and thus departmental spending. The studies were similar in that they all provided education to the leading surgeons within each department and one went further to provide a personalised ‘scorecard’ to assess costs compared to a baseline. However, despite the clear implication that merely providing education surrounding the topic improves cost efficiency, no survey was performed to ascertain baseline cost awareness [16-18].

Alongside increased financial education, pricing transparency regarding equipment costs can be used by trusts as an inexpensive way to reduce spending and has been recommended in previous studies as part of a cost-reduction strategy [9]. There are moves by NHS England such as the Getting It Right First Time national programme, which will use pricing transparency in equipment procurement [19]. Individual trusts can use this information to enable more cost-effective procurement to reduce spending. While this initiative is at the level of trust procurement of equipment and not for individual practitioners, individuals within trusts are likely to be involved in equipment procurement to fulfil the needs of their departments. Therefore, the move towards pricing transparency is a positive one in allowing practitioners to make informed choices.

While cost awareness is important for practitioners, keeping cost control as a priority may compromise the role of a clinician as the patient’s advocate [20]. Any strategy employed to reduce spending must not jeopardise the quality of patient care, and healthcare workers should not lose their autonomy to ensure the best clinical outcomes for patients. In anaesthetics, the need to be overprepared and cautious supersedes the need to save money and reduce waste, and using more equipment than needed can only be a secondary consideration. However, to sustainably empower professionals, education and price transparency can be important tools and are good initiatives when seeking cost‐efficiency improvements.

This study has shown there is support within the workforce towards a more sustainable practice regarding issues around the environmental impact of healthcare and reusable equipment. This is especially important within the anaesthetic speciality which contributes to a significant proportion of healthcare-associated pollution, with large amounts of equipment usage alongside waste anaesthetic gases [21]. Using reusable equipment can have financial benefits, for instance, when the application of reusable anaesthetic equipment compared to single-use was studied at an Australian hospital, there was a 46% decrease in annual financial cost when using reusable equipment [22]. However, in this study, they predicted that in Australia when using reusable equipment there was a higher carbon footprint generated, whereas in the United Kingdom/United States/Europe, the carbon footprint was lower. This reflects the different infrastructure within the countries and the dependence on different energy sources. Furthermore, cleaning reusable equipment may use more water than is required for manufacturing single-use equipment, therefore, the blanket notion that reusable equipment will be more environmentally friendly cannot necessarily be applied everywhere [22]. Infection control has led to hospitals embracing single-use anaesthetic equipment. However, there is no significant difference in infectious risk between single-use and reusable devices, with reusable devices having a better financial and environmental impact [23]. Therefore, if patient safety standards are maintained, using reusable equipment will enable a more cost-effective and environmentally friendly practice in the anaesthetic speciality.

The survey was freely available for respondents to complete to minimise selection bias and ensure randomisation; however, a limitation was that it was only sent to members of one anaesthetic department. While the NHS can create a homogenised experience for practitioners, different sites may have a different awareness of equipment costs due to regional training/education, therefore, this study would have missed this aspect. We hope that this study could provide a benchmark upon which future multi-centre (trust, regional, or national) research could be conducted. We believe that findings similar to ours would be uncovered, providing a greater database of research upon which to action reform. Furthermore, as with any freely available survey, those with stronger opinions may be more likely to respond, potentially biasing the responses. To combat this, the survey could have also asked about prior knowledge or training in health economics to assess if there was a significant improvement in the responses from those respondents. This study also focuses specifically on disposable anaesthetic equipment; however, the use of anaesthetic gases has become an important topic due to the financial and environmental impact of these substances. Broadening the scope of study to include drugs and anaesthetic gases would enable a more comprehensive view of the anaesthetic speciality [24].

## Conclusions

This research is part of a broad study into cost awareness among multiple specialities. Once completed, this should give an insight into cost awareness more widely within the NHS. Financial awareness is becoming a pressing issue due to the current financial burden within healthcare systems. We recommend that to enable better cost awareness health economics should be taught more widely at the undergraduate level. Price transparency should be standard practice in hospital departments, especially in anaesthesia where consumable use is high. Future research should then focus on the accessibility of this cost information and whether this information is used by healthcare workers to make a difference in their practice.

## Appendices

**Table 3:** Consumable items included in the anaesthetic survey.

1	One (1) 15-blade scalpel
2	One (1) 3-0 Vicryl (non-rapide) suture
3	One (1) 4-0 Prolene suture
4	One (1) 10 mL syringe
5	One (1) package of 2 4 × 4 sterile sponges
6	One (1) 1 L bag of intravenous normal saline
7	One (1) vial of 1.2 g intravenous co-amoxiclav
8	One (1) pair of standard sterile surgical gloves
9	One (1) box of fifty (50) surgical masks
10	One (1) disposable sterile surgical gown
11	One (1) size 4 transparent anaesthetic mask
12	One (1) size 8 cuffed oral endotracheal tube
13	One (1) size 4 disposable laryngoscope blade
14	One (1) disposable laryngoscope blade and handle
15	One (1) size 4 Guedel™ airway
16	One (1) disposable non-invasive blood pressure (NIBP) cuff
17	One (1) 50 mL plastic syringe
18	One (1) heat and moisture exchanger (HME) filter
19	One (1) spinal needle
